# The Roadmap of the Spleen: A Meta‐Analysis of Morphometric and Vascular Anatomy

**DOI:** 10.1002/hsr2.72667

**Published:** 2026-06-18

**Authors:** Ilias Miltiadis, Edgar S. Kafarov, Ali S. Dadashev, Pavel Burko, Daria A. Sukmanova, Nadezhda A. Stashevskaya, Oleg K. Zenin

**Affiliations:** ^1^ Department of Biomedicine Neuroscience and Advanced Diagnostics (BiND) University of Palermo Palermo Italy; ^2^ Department of Normal and Topographic Anatomy with Operative Surgery Kadyrov Chechen State University Grozny Russia; ^3^ Department of Biomedical Sciences Synergy University Moscow Russia; ^4^ Department of Human Anatomy Penza State University Penza Russia; ^5^ RUDN University Moscow Russia

**Keywords:** spleen anatomy, splenic artery, splenic vasculature, splenic vein

## Abstract

**Background and Aims:**

This systematic review aims to consolidate anatomical data on the spleen and its vasculature to aid in surgical planning and improve the understanding of splenic variability. The main research goal is to summarize reported ranges and pooled estimates for splenic dimensions and to map the common patterns and variations in the splenic vasculature.

**Methods:**

A systematic review and meta‐analysis were conducted, including 64 studies. The key parameters examined were the splenic length, width, thickness, and volume, and the branching patterns and origins of the splenic artery.

**Results:**

The analysis yielded approximate pooled mean estimates of splenic length of 10.47 cm, width of 6.92 cm, thickness of 3.55 cm, and volume of 219 cm^3^. In most cases, the splenic artery originates from the celiac trunk at the level of the 12th thoracic vertebra and follows a suprapancreatic course before branching into two or more vessels, each supplying distinct regions of the spleen.

**Conclusion:**

The spleen and its vascular network show considerable anatomical variability, with implications for surgical interventions. Accurate knowledge of these variations is essential for optimizing surgical outcomes and preserving splenic function.

## 1 Introduction

The spleen is one of the largest human lymphoid organs and plays a vital role in the immune system. The tissue of the spleen is abundantly vascularized, which makes it a surgically difficult site. Spleen tissue is virtually impossible to suture [1]. Given the rising incidence of traumatic injuries to the spleen, the development and implementation of organ‐preserving surgeries is urgently needed [2], and a thorough understanding of the anatomy of the spleen and upper abdominal cavity is needed. Because of its delicate structure and relatively exposed position in the abdominal cavity, the spleen is highly susceptible to damage during blunt force or penetrating trauma [3]. Splenic injury accounts for approximately a quarter of all abdominal injuries [4]. Surgery remains the leading treatment option in hemodynamically unstable patients or in patients with severe splenic injury. Operative treatment may include splenectomy or splenorrhaphy [5]. The intricacy and variability of the vessels at the splenic hilum add another layer of complexity to surgical interventions in this area. The region of the splenic hilum poses substantial challenges due to its confined surgical field, complicating the precise identification of vessels and successful laparoscopic operations. The challenge is further heightened by the difficulty in managing hemorrhage once vascular injury occurs, necessitating highly skilled surgical expertise for these procedures, and a thorough understanding of the anatomy of the spleen and its surrounding structures is essential [6, 7, 8]. The splenic artery, which supplies blood to the spleen, is especially challenging to characterize because of its variable length, tortuosity, relationship to the pancreas, branching pattern, and number of branches in the splenic hilum [9]. *This paper aims* to present available data on the organometric parameters of the spleen, such as length, width, thickness, and volume, as well as the peculiarities of the extra‐ and intra‐organ vasculature, both arterial and venous, in humans of various ages and sexes.

## 2 Materials and Methods

### 2.1 Study Design

This systematic review and meta‐analysis were conducted and reported in strict accordance with the Preferred Reporting Items for Systematic Reviews and Meta‐Analyses (PRISMA) statement guidelines [10]. A formal protocol was not registered prior to the initiation of this systematic review.

### 2.2 Search Strategy

A systematic literature search was conducted using the Scopus, Web of Science, and PubMed bibliographic databases up to March 9, 2024. We used the following query to find relevant articles: “(Splenic arter* OR splenic vein* OR splenic vessel* OR a* splenic* OR v* splenic* OR spleen)” AND “anatomy” AND “variation*.” We also checked the references of the retrieved papers for additional studies that were not captured by the search. The search was restricted to human studies published in English.

### 2.3 Study Selection

Two independent researchers screened the papers for eligibility. First, they excluded papers with irrelevant titles and abstracts. The authors subsequently reviewed the full texts of the remaining papers and applied the inclusion criteria. If there was any disagreement, a third researcher was consulted. Studies were included if they met the criteria of at least one of the researchers.

### 2.4 Study Quality Assessment

We used the anatomical quality assessment tool (AQUA), which was developed specifically for this purpose, to evaluate the quality of the selected studies [11, 12]. The AQUA tool assesses studies in five domains on a 20‐point scale (objectives and subject characteristics, study design, methodology, descriptive anatomy, and result reporting). Each question in the tool requires a “Yes,” “No,” or “Unclear” answer. Assuming that all questions for a domain have a “yes” answer, the risk of bias for that domain is considered “low.” Once a question had a “No” or “Unclear” answer, the domain was considered to have a “High” risk of bias. Each study was graded as high quality (all 5 domains at low risk of bias), medium quality (3–4 domains at low risk of bias), or low quality (0–2 domains at low risk of bias).

### 2.5 Statistical Analysis

The statistical analysis was performed via R, ver. 4.1 [13]. The meta R package was used to compute pooled parameters [14]. For continuous variables (e.g., spleen volume, length, and splenic artery diameter), pooled estimates and 95% confidence intervals (CIs) were calculated using a random‐effects model with the Restricted Maximum‐Likelihood estimator to account for anticipated between‐study heterogeneity. Only studies reporting continuous data as exact means and standard deviations (SD) or providing standard errors (SE) from which the SD could be derived ($\mathrm{SE}=\mathrm{SD}/\sqrt{N}$). Studies reporting only medians and interquartile ranges or absolute ranges of morphometric parameters were not included.

For the prevalence of anatomical variants (e.g., the number of primary splenic artery branches), a proportional random‐effects meta‐analysis was conducted. To appropriately stabilize variances and avoid out‐of‐bounds estimations for studies with proportions near 0 or 1, the Freeman–Tukey double arcsine transformation was applied prior to pooling.

Heterogeneity was evaluated using the Cochran *Q* test and quantified via the *I* ^2^ statistic. To explore the sources of high heterogeneity, predefined subgroup analyses were conducted based on measurement modality (e.g., CT, Ultrasound, Cadaveric). To ensure the robustness of the findings and address variations in study quality, sensitivity analyses (including stratification by quality assessment risk of bias) were performed to verify that low‐quality studies were not disproportionately driving the pooled estimates.

Small‐study effects and potential publication bias were initially assessed through the visual inspection of funnel plots. To address detected asymmetry, the Duval and Tweedie trim‐and‐fill method was implemented to impute theoretical missing studies and calculate an adjusted, bias‐corrected pooled estimate.

The results of the meta‐analysis for continuous variables are expressed as the pooled mean (*M*), SE, and 95% CI, whereas categorical variable results include percentages (%) or frequencies with 95% CIs.

## 3 Results and Discussion

**Figure 1:**
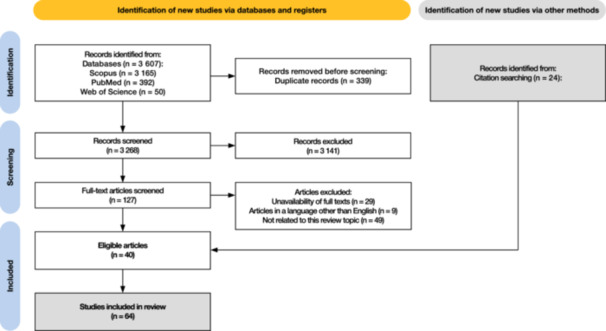
PRISMA flow chart of the selection of the studies in our review.

The initial search yielded 3607 publications that met the search query criteria, from which 339 duplicates were excluded. After the initial screening by two independent researchers, 3141 papers were excluded. The screening of the full‐text materials was subsequently conducted, which resulted in the exclusion of 87 articles (Figure 1). Thus, 40 publications met the inclusion criteria. The bibliographic lists of the included studies were also searched, resulting in 24 papers. In total, 64 studies were included in this review (Table 1, Supporting Information S5: Table S1).

**Table 1:** Summary of studies included in the review.

Parameter	*n*	Percentage (%)
Modality		
CT	22	34.4
Cadaveric	22	34.4
Ultrasound	8	12.5
Corrosion casting	6	9.4
Laparoscopy	2	3.1
MRI	1	1.6
Mixed/Multiple	3	4.6
Geographic region		
Asia	26	40.7
Europe	23	35.9
North America	7	10.9
Africa	4	6.2
South America	3	4.7
Oceania	1	1.6

### 3.1 Quality Assessment of the Included Articles

In the first domain, which evaluates the clarity and appropriateness of the study objective(s) and the subject sample(s), most studies have clearly defined objectives and appropriate subject samples. However, 45 studies had a high risk of bias in this domain. In the second domain, which assesses the suitability and validity of the study design for addressing the research question(s), most studies had appropriate and widely accepted study designs. Only three studies had a high risk of bias in this domain. The third domain evaluates the description and reproducibility of the methods/techniques applied in the study. Here, most studies (60 out of 64) were considered high risk. The fourth domain assesses the accuracy and clarity of the description of anatomy in the study. Twenty‐seven studies provided accurate and clear descriptions of anatomy, but 37 studies had issues such as inaccurate or unclear descriptions of anatomy or anatomical observations that were not clearly explained. The fifth domain evaluates the comprehensibility and consistency of the results reported in the study. Twenty‐two studies had clear and consistent results, but 42 studies had issues such as unclear or incomprehensible reporting of results or reported values that were inconsistent or did not correspond to the number of subjects in the study. Finally, 62% of the studies received a “low” quality assessment, 36% received a “medium” quality assessment, and 2% achieved a “high” quality assessment.

### 3.2 Spleen Organometry

**Figure 2:**
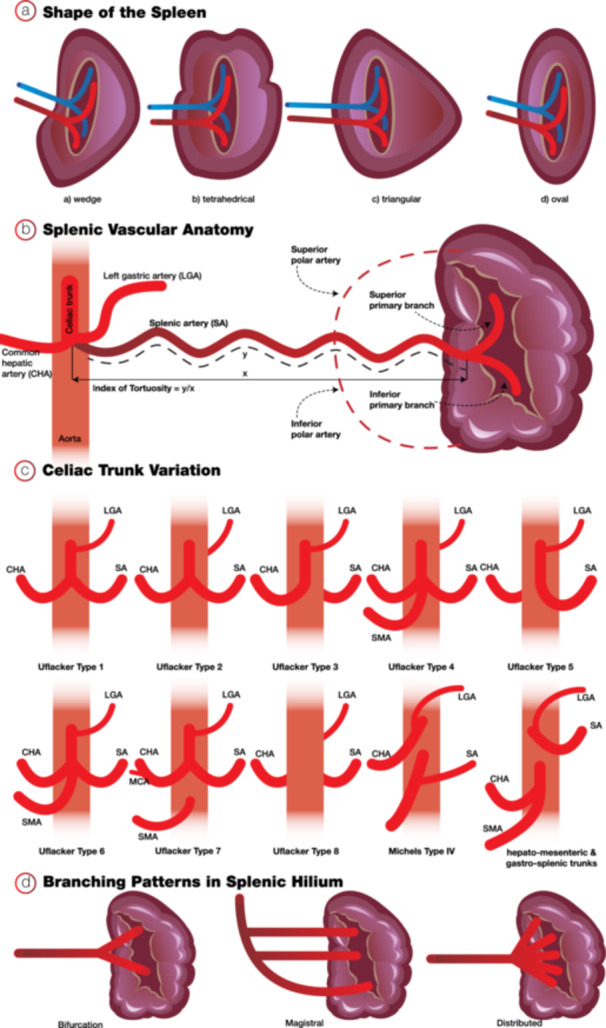
Spleen: (a) shape, (b) arterial vasculature, (c) celiac trunk variation, and (d) splenic artery branching pattern.

The spleen has an average length of 9.58 ± 1.22 cm according to ultrasound examination of the spleen carried out on 43 adults [15]. Singh et al. [16] investigated the correlation between body length and spleen length. This study included 160 adults—80 men and 80 women. Thus, in the male group with heights ranging from 151 to 155 cm (*N* = 12), the mean spleen length was 9.29 ± 1.056 cm; in the 156–160 cm group (*N* = 13), it was 9.565 ± 1.564 cm; in the 161–165 cm group, it was 9.877 ± 1.507 cm (*N* = 18); in the 166–170 group (*N* = 21), it was 10.134 ± 1.415 cm; and in the 171–175 group (*N* = 16), it was 10.706 ± 0.82 cm. For women with a height of 146–150 cm (*Ν* = 14), the mean spleen length was 8.828 ± 1.461 cm; for women with a height of 151–155 cm (*Ν* = 22), it was 8.945 ± 1.483 cm; for women with a height of 156–160 cm (*Ν* = 16), it was 9.189 ± 1.393 cm; for women with a height of 161–165 cm (*Ν* = 12), it was 9.464 ± 1.058 cm; and for women with a height of 165–170 cm (*Ν* = 16), it was 9.898 ± 1.261 cm. It is therefore likely that spleen length increases as body length increases. However, it should be noted that there were noticeably fewer females than males in the corresponding height groups. The main drawback of this study is the lack of correlation analysis between the investigated parameters. According to a CT imaging study of 90 spleens, the length of the spleen was 11.17 ± 1.74 cm, and the width was 9.85 ± 1.56 cm [17]. In 2015, researchers from Türkiye noted the difference in morphometric parameters according to sex: in women, the average width of the spleen was 7.58 ± 1.56 cm, the length was 9.87 ± 1.28 cm, and the thickness was 3.34 ± 0.79 cm; in men, the width was 8.75 ± 1.84 cm, the length was 11.01 ± 1.186 cm, and the thickness was 4.12 ± 1.09 cm [18]. These findings are consistent with the data obtained by the Greek research group: spleen length was significantly greater in men (12.18 ± 2.2 cm) than in women (10.71 ± 1.6 cm) (*p* < 0.05). Overall, the spleen is 11.69 ± 2.1 cm long, 7.54 ± 1.6 cm wide, and 4.20 ± 1.3 cm thick [19]. Khaleel et al. (2021) reported the mean cadaveric dimensions of the spleen as 11.64 cm (length), 7.3 cm (width), and 3.6 cm (thickness) [20]. An Iranian cadaveric study based on 693 samples revealed a splenic width of 0.5–22 cm with an average of 11.32 cm, a length of 0.5–15 cm with an average of 8.05 cm, and a thickness of 0.05–9.5 cm with an average of 2.01 cm [21]. Jagdish and Ashoka noted the presence of wedge‐shaped (43.3%), tetrahedral (28.3%), triangular (15%), and oval (2%) spleens in 60 cadavers (Figure 2a). The following parameters were also measured: length, 10.5 ± 2.236 cm; width, 4.25 ± 1.513 cm [22]. According to an ultrasound study performed in Pakistan, the mean length of the spleen was 9.76 ± 1.37 cm, and the width was 5.15 ± 1.42 cm [23].

The random‐effects pooled mean for splenic length was 10.47 cm (95% CI: 9.79–11.16; *I* ^2^ = 96.4%) (Supporting Information S1: Figure S1). Subgroup analysis by modality revealed significant variation (*p* < 0.0001), with CT imaging yielding the largest mean length (11.17 cm) compared to cadaveric studies (10.73 cm) and ultrasound (9.72 cm). A quality sensitivity analysis demonstrated no significant difference (*p* = 0.85) between medium‐quality (10.53 cm) and low‐quality (10.34 cm) studies. The pooled mean width was 6.92 cm (95% CI: 5.30–8.53; *I* ^2^ = 99.1%), with significant heterogeneity driven by modality (*p* < 0.0001); CT reported the widest dimension (9.85 cm) versus ultrasound (5.15 cm). The overall pooled thickness was 3.55 cm (95% CI: 2.02–5.08) (Table 2).

**Table 2:** Length, width, and thickness of the spleen.

Study	*N*	Mean (*Μ*)	Standard deviation (SD)	Standard error (SE)
Splenic length (cm)				
Li et al. [15]	43	9.58	1.22	0.19
Studer et al. [17]	22	11.17	1.74	0.37
Michalinos et al. [19]	50	11.69	2.1	0.30
Mohammadi et al. [21]	693	11.32	3.1	0.12
Jagdish and Ashoka [22]	60	10.5	2.24	0.29
Muhammad et al. [23]	200	9.76	1.42	0.10
Covantsev et al. [24]	330	9.43	3.62	0.20
Splenic width (cm)				
Studer et al. [17]	22	9.85	1.56	0.33
Michalinos et al. [19]	50	7.54	1.6	0.23
Mohammadi et al. [21]	693	8.05	2.35	0.09
Jagdish et al. [22]	60	4.25	1.51	0.2
Muhammad et al. [23]	200	5.15	1.42	0.10
Covantsev et al. [24]	330	6.71	2.72	0.15
Splenic thickness (cm)				
Michalinos et al. [19]	50	4.2	1.3	0.18
Mohammadi et al. [21]	693	2.01	0.9	0.03
Muhammad et al. [23]	200	4.46	1.1	0.08

Abbreviation: *N*, sample size.

### 3.3 Spleen Volume

Prassopoulos et al. [25] reported that the organometric values of the normal spleen do not significantly correlate with age, body length and weight, body mass index, or transverse diameter of the first lumbar vertebra. The mean volume of the spleen was 214.6 cm^3^ (107.2–341.5 cm^3^). Its value did not differ according to age or sex: in men, it was 215.1 cm^3^; in women, it was 214.0 cm^3^. In their ultrasound study, Loftus et al. [26] reported that the mean volume of the spleen was 110 ± 70 cm^3^. A CT study of 11 adults revealed that the splenic volume was 219 ± 76 cm^3^ [27]. Hoefs et al. [28] reported 201 ± 77 cm^3^. Yetter et al. [29] reported contrasting data from CT scans of 142 adults and reported that the spleen volume was 512.6 ± 349.1 cm^3^. The mean spleen volume of 23 healthy adults was 185 ± 65 cm^3^ according to the CT scans of Chen et al. [30]. Studer et al. [17] measured 224.9 ± 91.5 cm^3^; moreover, they noted significant variability in the value of this parameter across different sex groups (244.4 ± 80.9 cm^3^ in men and 205.4 ± 98 cm^3^ in women [*p* < 0.05]). Researchers came to a similar conclusion on the basis of ultrasound scanning data: the spleen volume was 136.05 ± 61.14 cm^3^ in women and −220.7 ± 115.35 cm^3^ in men [18]. Using CT scans of 230 healthy adults in Japan, Harris et al. [31] reported a mean volume of 127.4 ± 62.9 cm^3^ for this organ. On the basis of 45 contrast‐enhanced CT studies, Linguraru et al. [32] noted that the volume of the spleen was 236.89 ± 77.58 cm^3^. According to a study of 200 healthy women in Pakistan, the mean spleen volume was 148.5 ± 82.3 cm^3^ [23] (Table 3).

**Table 3:** Spleen volume.

Study	*N*	Volume of the spleen (cm^3^)		
		Mean (*Μ*)	Standard deviation (SD)	Standard error (SE)
Henderson et al. [27]	11	219	76	22.92
Loftus et al. [26]	30	110	70	12.78
Hoefs et al. [28]	11	201	77	23.22
Yetter et al. [29]	142	512.6	349.1	29.3
Chen et al. [30]	23	185	65	13.55
Harris et al. [31]	230	127.4	62.9	4.15
Linguraru et al. [32]	45	236.89	77.58	11.56
Studer et al. [17]	22	224.9	91.5	19.50
Cruz‐Romero et al. [33]	101	244.4	116.4	11.58
Muhammad et al. [23]	200	148.5	82.3	5.82

Abbreviation: *N*, sample size.

The overall pooled mean splenic volume was 219.20 cm^3^ (95% CI: 151.56–286.85; *I* ^2^ = 97.4%) (Supporting Information S2: Figure S2). As with linear dimensions, modality significantly influenced the estimates (*p* < 0.0001), with CT imaging yielding a substantially higher pooled volume (250.69 cm^3^) compared to a single cadaveric study (110.00 cm^3^). After stratifying by study quality, medium‐quality studies reported a larger pooled volume (254.65 cm^3^) than low‐quality studies (196.49 cm^3^), although the difference was not statistically significant (*p* = 0.52).

A continuous meta‐analysis comparing spleen volumes between sexes revealed that male spleens were significantly larger than female spleens, with a pooled mean difference of 69.98 cm^3^ (95% CI: 38.78–101.17; *p* < 0.0001; *I* ^2^ = 51.4%) (Table 4).

**Table 4:** Volume of the spleen in the sex groups.

Study	Males			Females		
	*N*	*M* (cm^3^)	SD (cm^3^)	*N*	*M* (cm^3^)	SD (cm^3^)
Çeliktas et al. [18]	72	220.7	115.35	78	136.05	61.14
Studer et al. [17]	45	244.4	98	45	205.4	80.9
Cruz‐Romero et al. [33]	32	304.3	119.6	69	216.6	104.5

Abbreviation: *N*, sample size.

The significant heterogeneity (*I* ^2^ > 97%) observed across organometric parameters is heavily driven by the measurement modality. Throughout our subgroup analyses, CT imaging consistently yielded larger dimensions and volumes compared to ultrasound and cadaveric measurements. This variance is physiologically and methodologically expected. In‐vivo CT imaging captures the spleen in its natural, blood‐filled hemodynamic state, accurately reflecting its true 3D volume. In contrast, cadaveric measurements are subject to post‐mortem exsanguination and tissue shrinkage during fixation, leading to substantially lower volume estimates. Furthermore, ultrasound measurements can introduce operator‐dependent variability and often rely on 2D linear extrapolations that may underestimate the true volume of an irregularly shaped organ. Therefore, the wide variability reported across the literature—such as volumes likely reflects these fundamental methodological differences rather than extreme physiological anomalies. While splenic dimensions are strongly influenced by modality, study population, sex, body habitus, and postmortem changes, pooling these parameters provides a valuable macro‐level overview of the dimensions encountered across diverse diagnostic settings. Consequently, the pooled values presented herein are not intended to serve as definitive normal reference standards. Rather, they function as modality‐dependent approximate estimates that can help anticipate expected ranges when interpreting different types of medical imaging or performing cadaveric dissections.

### 3.4 Splenic Vasculature

Early cadaveric dissections and corrosion casting techniques were instrumental in mapping the initial morphological blueprints of the splenic vasculature, establishing the primary classifications of hilar branching, celiac trunk variations, and segmental distribution. Today, these historical anatomical frameworks remain highly relevant, serving as the essential interpretive baseline for contemporary radiological workflows. Modern imaging modalities, particularly contrast‐enhanced MDCT and three‐dimensional volume rendering, now allow clinicians to visualize these established anatomical variants *in vivo* with millimeter precision. Consequently, the historical anatomical norms cataloged in older literature do not represent obsolete data; rather, they provide the necessary structural vocabulary that enables surgeons and radiologists to accurately interpret modern preoperative virtual mapping, thereby facilitating safer interventions.

#### 3.4.1 Arterial Extra‐Organ Vasculature

Conventionally, the splenic artery, along with the left gastric and common hepatic arteries, is a branch of the celiac trunk, the first branch of the abdominal aorta [34] (Figure 2b). These arteries may branch off separately or form a common hepatosplenic trunk, with the common hepatic and splenic arteries serving as branches. Many classifications of celiac trunk variations have been proposed, the most common of which is the Uflacker classification (1997) (Figure 2c). According to this classification, eight types of celiac trunk branching were distinguished (Table 5). Other researchers, namely Lipshutz [35], classified these variations into four types; Adachi [36], with six types; Morita [37], with five types; and Michels [38], with six types. Moreover, more recent attempts were made by Acar et al. [39] and Türkyılmaz et al. [40], who combined the celiac trunk and hepatic artery.

**Table 5:** Celiac trunk classification.

Type by Uflacker classification (1997)	Description
Type 1 – Trifurcation Classical	Common hepatic, splenic, and left gastric arteries branch out of the celiac trunk at one point.
Type 1 – Trifurcation Nonclassical	Common hepatic and splenic arteries branch out of the celiac trunk at one point; the site of the left gastric artery varies.
Type 2 – Hepato‐splenic trunk	Common hepatic and splenic arteries make up the common trunk, whereas the left gastric artery branches out of the aorta separately.
Type 3 – Hepato‐gastric trunk	Common hepatic and left gastric arteries make up a common trunk, whereas the splenic artery branches out of the aorta separately.
Type 4 – Hepato‐spleno‐mesenteric trunk	Common hepatic, splenic, and superior mesenteric arteries make up the common trunk, whereas the left gastric artery branches out of the aorta separately.
Type 5 – Gastro‐splenic trunk	Left gastric and splenic arteries make up a common trunk, whereas the common hepatic artery branches out of the aorta separately.
Type 6 – Celiaco‐mesenteric trunk	The celiac trunk and the superior mesenteric artery comprise a common trunk.
Type 7 – Celiaco‐colic trunk	The middle colic artery and the celiac trunk comprise a common trunk.
Type 8 – Absence of celiac trunk	Celiac trunk is absent, meanwhile common hepatic, splenic and left gastric arteries branch out of aorta directly.

During embryogenesis, the paired dorsal aorta originates from the ventral segmental arteries. Fusion of the dorsal aorta to a single aortic trunk leads to an odd number of ventral segmental arteries connected by longitudinal anastomosis. The left gastric artery, splenic artery, common hepatic artery, superior mesenteric artery, and inferior mesenteric artery originate from the 10th, 11th, 12th, 13th, and 19th ventral segmental arteries, respectively. Regression of the proximal portion of the 11th and 12th ventral segmental arteries and the intersegmental anastomosis connecting the 12th to the 13th results in the formation of the celiac trunk and the superior mesenteric artery [42].

Much of the literature on celiac trunk branching concludes that the most common type 1 branching is trifurcation (Table 6). The origin of the splenic artery is most often at the level of the 12th thoracic vertebra (Table 7). Ekingen et al. [48] reported a classic variant of splenic artery branching in 79.47% of cases; in most cases, this variant was a branch of the bifurcation of the celiac trunk (68%), trifurcation in 10.94% of cases, and quadrifurcation in 3% of cases [48].

**Table 6:** Celiac trunk variations.

Study	*N*	Type by Uflacker classification (1997), *n*								Other, *n*
		1	2	3	4	5	6	7	8	
Torres et al. [43]	1569	1455	34	4	0	64	8	0	2	
Araujo‐Neto et al. [44]	60	54	5	1	0	0	0	0	0	
Osman and Abdrabou [45]	1000	905	28	6	0	43	6	0	10	2 (IV according to Michels Classification), 2 non‐classified
Laleye et al. [46]	200	187	2	0	1	5	2	0	1	2 (IV according to Michels Classification)
Türkyılmaz et al. [41]	941	856	22		5	12	6			20 (hepato‐mesenteric and gastro‐splenic trunks) 22 (not classified)

Abbreviation: *N*, sample size.

**Table 7:** Splenic artery origin.

Study	*N*	Vertebrae, *n*								Not stated, *n*
		T10	T11	T11–12	T12	T12–L1	L1	L1–L2	L2	
Pinal‐Garcia et al. [47]	140	1	8	5	67	19	40			
Türkyılmaz et al. [41]	941		3	13	427	230	247	12	2	7

Abbreviation: *N*, sample size.

In most cases (74.1%), the splenic artery has a suprapancreatic course; in 18.4% of cases, the splenic artery passes in front of the pancreas; in 4.6% of cases, the splenic artery has an intrapancreatic course; and in 2.8% of cases, the splenic artery passes behind the pancreas [49]. A recent study by Covantsev et al. [24] reported that the splenic artery is located suprapancreatically in 70.3% of cases, anterior to the pancreas in 4.85% of cases, within the parenchyma of the pancreas in 14.85% of cases, and posterior to the pancreas in 10% of cases [24]. Zheng et al. highlighted several variants of the intrapancreatic course of the splenic artery. In 66.6% of the patients, the middle part of the splenic artery had either a retro‐ or intrapancreatic course, and the distal part had such a course in 4.1% of patients; in 1.9% of patients, the distal ¾ of the splenic artery went through the pancreatic parenchyma or behind it [8] (Table 8).

**Table 8:** Course of the splenic artery relative to the pancreas.

Study	*N*	Course of splenic artery, *n*			
		SP	IP	RP	AP
Pandey et al. [49]	320	237	15	9	59
Xu et al. [50]	48	19	29		
Xu et al. [51]	36	16	20		
Gangadhara and Hemasankar [52]	30	19		11	
Zheng et al. [8]	317	87	230		
Meet et al. [53]	15	14		1	
Zhu et al. [54]	169	62	24	83*	
Covantsev et al. [24]	330	230	49	33	16

Abbreviations: AP, anteriopancreatic; IP, intrapancreatic; *N*, sample size; RP, retropancreatic; SP, suprapancreatic.

* Horizontal axis of the pancreas.

Inoko et al. [55] identified two types of splenic artery courses: type A, in which the splenic artery is tortuous and passes above the pancreas; and type B, in which the splenic artery passes relatively straight to the dorsal face of the pancreas. Their data revealed that type A was observed in 66.6% of patients and that type B was observed in 33.4% of patients. Wada et al. [56], in their laparoscopic distal pancreatectomy study, reported 83% and 17% of patients with type A and type B disease, respectively. Sylvester et al. [57] proposed the splenic artery tortuosity index, which is defined as the ratio of the artery's total length to the direct distance between its origin and end (Figure 2b). The higher the index is, the greater the tortuosity of the artery. The results revealed significant variability, ranging from 1.01 (almost straight artery) to 5.25 (very tortuous artery).

Approaching the hilum, the splenic artery divides into smaller terminal arteries (Table 9). There are several types of branching: bifurcational, magistral, and distributed (Figure 2d). Bifurcational branching is the most universally recognized vascular topology, where the parent artery divides symmetrically into exactly two primary daughter vessels. Magistral branching (often referred to as a “truncal” or “ladder‐like” pattern) is designed for long‐distance, low‐resistance linear perfusion. In this configuration, the main arterial trunk maintains a relatively constant, large diameter as it courses toward the spleen, giving off smaller secondary collateral branches along its path [49, 60]. The primary division of the main splenic trunk is significantly delayed, typically occurring within 1 to 2 cm of the splenic hilus. Lying morphologically opposite to the magistral design is distributed branching, which is defined by the early, o ften premature termination of the main vascular trunk. In this configuration, the artery divides into multiple, relatively equivalent branches that disperse broadly into the target tissue long before reaching the anatomical hilum of the organ [24]. Notably, this conceptual framework of magistral and distributed architecture is deeply rooted in the work of Viktor N. Shevkunenko, who categorized visceral vascular architecture into magistral, mixed, and distributed patterns, which remains highly relevant in contemporary three‐dimensional modeling and computed tomography studies of the abdomen [61]. Rarely, the splenic artery does not branch out but rather enters the spleen parenchyma in the hilum as a single vessel.

**Table 9:** Types of splenic artery branching.

Study	*N*	Types of branching, *n*			
		No branching	Bifurcation	Magistral	Distributed
Pandey et al. [49]	320	9	311		
Xu et al. [50]	48			18	38
Xu et al. [51]	36			13	23
Zheng et al. [8]	317			205	112
Ashok and Kiran [58]	67	8		17	42
Sundar and Sangeetha [59]	60	10		14	36
Covantsev et al. [24]	330	11	134	66	119

Abbreviation: *N*, sample size.

A cadaveric study revealed that the diameter of the splenic artery was 6.44 ± 1.4 mm, and a notable positive correlation between the artery diameter and organ weight was also noted [19]. The length of the splenic artery, according to Brinkman et al. [62], ranges from 165 to 225 mm. Table 10 shows the splenic artery length and diameter data on the basis of the morphometric results of different authors.

**Table 10:** Diameter and length of the splenic artery.

Study	*N*	Mean (*Μ*)	Standard deviation (SD)	Standard error (SE)
Diameter of splenic artery (mm)				
Delahunt et al. [63]*	20	7.1	1.5	0.34
Geelkerken et al. [64]	14	6.1	1.4	0.37
Kirbas et al. [65]	8	5.3	0.4	0.14
Silveira et al. [66]	30	5.3	0.3	0.05
Malnar et al. [67]	90	6.1	0.5	0.05
Michalinos et al. [19]	50	6.44	1.4	0.2
Saldarriaga et al. [68]	26	5.9	1.0	0.2
Length of splenic artery (mm)				
Fataftah et al. [69]	219	94.1	13.3	0.9
Saldarriaga et al. [68]	26	92.5	16.9	3.31

Abbreviation: *N*, sample size.

* Intraabdominal ultrasound.

The overall pooled mean splenic artery diameter was 5.99 mm (95% CI: 5.53–6.45) (Supporting Information S3: Figure S3). A sensitivity analysis stratifying the data by study quality revealed no significant difference (*p* = 0.57) between Low‐quality studies (5.93 mm) and Medium‐quality studies (6.17 mm), indicating that the overall pooled estimate was not disproportionately skewed by studies with a higher risk of bias. Visual inspection of the funnel plot suggested potential asymmetry favoring the publication of larger arterial diameters. Subsequent application of the Duval and Tweedie trim‐and‐fill method imputed three theoretical missing studies, yielding a more conservative, bias‐adjusted pooled mean diameter of 5.47 mm (95% CI: 4.82–6.11). Due to insufficient data, a meta‐analysis of splenic artery length could not be performed.

#### 3.4.2 Arterial Organ Vasculature

The first serious discussions and analyses of splenic intra‐organ vasculature organization and segmentation were articulated by the French anatomist Assolant [70]. Assolant suggested that each artery entering the splenic hilum supplies blood to a parenchymatous “compartment” that is completely independent of the others. The term “parenchymal units” was used to describe these compartments.

The corrosion casting method for parenchymatous organs has led to a new round of studies on the intra‐organ arterial vasculature, including the spleen. Huu et al. [71] suggested that the spleen is composed of autonomous vascular segments supplied by blood vessels that can be ligated in the hilum. These segments overlap perpendicularly to one another on top of the main axis of the organ and are clearly separated from each other by avascular or low‐vascularity areas. Simionescu et al. [72] used the term *lobus* for these sections. The authors defined the upper lobe (*lobus lienalis superior*) and lower lobe (*lobus lienalis inferior*), each consisting of two segments: the polar (*segmentum polare*) and mesolienal (*segmentum mesolienale*) segments.

Cortés and Gómez Pellico [73] distinguished three types of splenic artery branching: those located at the caudal end of the pancreas (33.3%), those located between the caudal end and the splenic hilum (40%), and those located directly in the splenic hilum (26%). In 27 cases (90%), the splenic artery split into two branches, the upper and lower lobular arteries, forming two independent supply regions with a larger volume of the upper artery. These regions are separated from each other by a vascular‐free zone located in a transversely oblique direction. The upper lobar artery was divided intraparenchymally into two branches in 36.6% of cases, into three branches in 33.3% of cases, into four branches in 26.6% of cases, and into five branches in 3.3% of cases. The inferior lobar artery had two branches in 83.3% of cases, three branches in 10% of cases, and four branches in 6.6% of cases. The division of the upper and lower lobar arteries yielded the splenic segments, which ranged from 5 to 8.

The findings of García‐Porrero, Lemes [74] confirm the findings of previous studies: in 92.82% of cases, the splenic artery was divided into two branches—upper and lower—and in 7.18% of cases, the splenic artery was divided into three branches—upper, middle, and lower. The authors emphasized statistically significant differences in the frequency of the three branches in the different sex groups: 3.76% and 16.76% in males and females, respectively (*p* < 0.001). This may be due to differences in sample size: 133 men and 48 women were included in the study. Researchers have also identified the polar arteries. The term polar artery refers to a vessel that enters one of the poles of the spleen outside the hilum regardless of its origin [39]. The upper polar artery was found in 29.28% of the patients; most often, it was a branch of the splenic artery trunk. The inferior polar artery was found in 44.75% of the patients; in most patients, it originated from the left gastric artery (37.04%) or splenic artery trunk (29.63%). In 10.49% of the patients, both the superior and inferior polar arteries were present. Daisy Sahni et al. [75] reported that a superior pole artery branches from the splenic artery after the posterior gastric artery branch and does not reach 4–5 cm to the splenic hilum in 51% of cases, whereas 86.5% of cases had an inferior polar artery branching from the lower lobal artery of the spleen. Ishikawa et al. [76] noted the superior polar artery in 59% of cases, which gave off one or more gastric branches in 45% of cases. Treutner et al. [77] noted the predominance of the bifurcation division of the splenic artery (93.8%). Four segments were most common (81.3%), whereas three and two segments accounted for 15.6% and 3.1%, respectively, of the cases. As shown in Table 11, the number of splenic segments observed varied across studies.

**Table 11:** Number of splenic segments.

Study	*N*	Number of segments, *n*						
		2	3	4	5	6	7	8
Gupta et al. [76]	50	42	8					
Cortés and Gómez Pellico [73]	30				8	16	4	2
Treutner et al. [77]	32	1	5	26				
Meet et al. [53]	15	1		7	4	3		

Abbreviation: *N*, sample size.

The primary branch arises from the splenic artery division, penetrating the spleen parenchyma and giving rise to segmental branches [79]. The term is synonymous with “lobal branch” [73]. This definition shares a meaning with the term “zonal arterial segment” of the renal intra‐organ arterial vasculature [80]. According to Ignjatovic, Bergamaschi [81], the diameter of the superior primary branch ranges from 2.2 to 5.4 mm (mean 3.4 mm), and that of the inferior branch ranges from 1.3 to 4.6 mm (mean 2.8 mm); in the case of three primary branches, the diameter of the middle branch ranges from 1.1 to 3.4 mm (mean 2.2 mm). The length of the inferior branch from the bifurcation of the splenic artery to the first division ranged from 12 to 60 mm (mean 36 mm). Most often, the inferior branch is split into two branches (70.6% of cases); in 27.4% of cases, it is split into three branches; and in 1% of cases, it is divided into four or five branches (Table 12).

**Table 12:** Number of primary branches of the splenic artery.

Study	*N*	Number of primary splenic artery branches, *n*					
		1	2	3	4	5	6
Gupta et al. [78]	50		42	8			
Mikhail et al. [82]	25		19	6			
Katritsis et al. [79]	70		60	10			
Cortés and Gómez Pellico [73]	30		27	3			
García‐Porrero and Lemes [74]	181		168	13			
Sow et al. [83]	100		84	16			
Treutner et al. [77]	32		30	2			
Ignjatovic and Bergamaschi [81]	120		93	9			
Chaware et al. [84]	111		95	16			
Swamy et al. [85]	60		40	10	10		
Londhe [86]	50		45	5			
Zheng et al. [8]	317	22	250	43	2		
Ishikawa et al. [76]	104		96	8			
Shwetha and Dakshayani [87]	79		56	19	4		
Covantsev et al. [24]	330	11	273	32	10	2	2

Abbreviation: *N*, sample size.

In a recent study from India, which focused on the morphometry of primary branches of the splenic artery, the diameter of the upper branch was 2.2 ± 0.82 mm, the length was 1.7 ± 0.74 cm, and the diameter and length of the lower branch were 2.1 ± 0.81 mm and 1.92 ± 0.98 cm, respectively. In the trifurcation division of the splenic artery, the diameter of the resulting middle branch is 2.08 ± 0.82 mm, and the length is 1.13 ± 0.66 cm [87].

A proportional meta‐analysis assessing the anatomical variation of the splenic artery revealed that a two‐branch configuration is the most frequently observed anatomical pattern (Supporting Information S4: Figure S4). The pooled global prevalence of exactly two primary branches was 84.0% (95% CI: 79.9%–87.8%; *I* ^2^ = 73.6%). Conversely, the prevalence of exactly three primary branches was substantially lower at 12.1% (95% CI: 9.6%–14.8%; *I* ^2^ = 50.2%). Subgroup analyses indicated that measurement modality significantly influenced the reported prevalence of both two‐branch (*p* < 0.0001) and three‐branch (*p* = 0.033) configurations, whereas study quality did not significantly alter the findings.

#### 3.4.3 Venous Extra‐Organ Vasculature

The splenic vein is formed by the confluence of intra‐organ venous branches of the spleen into a single vessel in the hilum. The tributaries of the splenic vein may be the inferior and accessory mesenteric veins, which subsequently merge with the superior mesenteric vein to form the portal vein. Krumm et al. [88] proposed a classification system for the splenic vein and other tributaries of the portal vein in which 10 variants were distinguished. The authors reported that the three most common variants were as follows: (1) the splenic vein takes the inferior mesenteric vein and merges with the superior mesenteric vein to form the portal vein; (2) the splenic, inferior, and superior mesenteric veins merge to form the portal vein at a single point; and (3) the inferior mesenteric vein merges into the superior mesenteric vein, which forms the portal vein when united with the splenic vein. These variants accounted for 85.6% of all the cases, with the remaining seven variants occurring less frequently (14.4%). The mean diameter of the splenic vein ranges from 4.4 to 18 mm, with a median value of 10.2 mm [88]. Russkikh [89] reported that the mean length of the splenic vein was 125 mm, with a diameter of 7.5 mm.

#### 3.4.4 Venous Intra‐Organ Vasculature

Only one paper on the splenic intra‐organ venous vasculature was identified during the systematic search. The spleen is divided into two major venous territories or lobes separated by an avascular plane. The lobes, for their part, are divided into segments separated from each other by avascular zones. The number of segments varies from four to eight, with an average of five. Gómez Pellico et al. [90] distinguished four types of splenic vein formation according to the number and characteristics of the veins that merged into the vein. The most common type I splenic vein is formed from two wide venous trunks, each draining the respective halves of the spleen. The other types are characterized by the presence of polar segmental veins that also drain into the splenic vein.

### 3.5 Certainty of Evidence

While our sensitivity analyses demonstrated no statistically significant differences in pooled estimates between low‐ and medium‐quality studies, the overall high risk of bias across the literature necessitates a cautious interpretation of these findings. With 62% of the studies included classified as low quality, predominantly due to unclear methodological descriptions and inconsistent result reporting, the overall certainty of evidence for these anatomical values remains moderate to low. The pooled estimates generated in this meta‐analysis provide a useful aggregate approximation that does not appear to be artificially skewed by the lowest‐quality data. However, due to the widespread methodological inconsistencies in the primary literature, these dimensions and anatomical variations should be viewed as reliable approximations rather than absolute anatomical laws. Future anatomical studies must adhere strictly to standardized reporting guidelines, such as the AQUA checklist [12], to elevate the certainty of anatomical evidence and improve confidence in surgical mapping.

### 3.6 Clinical Implications

The anatomical data synthesized in this meta‐analysis carry direct operational consequences for both open and minimally invasive abdominal procedures.

The confirmation that a two‐branch primary arterial configuration is the most prevalent anatomical variant (84.0%) provides a practical morphological basis for spleen‐salvaging procedures. Because these primary branches supply relatively autonomous parenchymal compartments separated by avascular planes, targeted ligation of either the superior or inferior primary branch allows for highly controlled segmental dearterialization. This anatomical predictability is vital for optimizing tissue preservation during trauma management.

Isolating splenic vessels from the pancreatic parenchyma is the primary technical hurdle in distal pancreatectomy with splenic preservation. Our data highlights why this can be treacherous: while the artery is usually suprapancreatic, it runs directly anterior to, posterior to, or entirely within the pancreatic parenchyma in nearly 30% of cases. Furthermore, high arterial tortuosity (Type A configuration) necessitates attention to avoid vascular injury and subsequent ischemia or hemorrhage.

Failure to recognize splenic vascular anomalies is a primary driver of intraoperative hemorrhage. The high prevalence of polar arteries entering the spleen outside the main hilum requires strict attention. An unrecognized superior polar artery (present in up to 59% of cases) or inferior polar artery (present in up to 86.5% of cases) could easily be avulsed during the initial mobilization of the splenic ligaments.

## 4 Conclusions

This paper provides a systematic review and meta‐analysis of anatomical studies on the spleen and splenic vessels (arteries and veins). The morphometric parameters of the spleen (length, width, thickness, and volume) and splenic vessels, as well as variants of their branching (arteries) and merging (veins), were analyzed.

The results of the meta‐analysis indicate that approximate pooled mean estimates of length, width, and volume of the spleen are 10.47 cm, 6.92 cm, and 219 cm^3^, respectively. Researchers have noted a tendency for the length of the spleen to increase as body length increases. The spleen volume was also significantly different between the two sex groups, with 69.98 cm^3^ more in men (254.20 cm^3^, SE: 23.9; 95% CI: 207.3; 301.01 cm^3^) than in women (185.11 cm^3^, SE: 25.6; 95% CI: 134.93; 235.3 cm^3^). However, given the high statistical heterogeneity and the varied methodological quality of the included studies, these values should be viewed as standard or normal reference values. Instead, they represent modality‐dependent approximate estimates, as splenic dimensions are strongly influenced by the measurement technique, population demographics, and postmortem changes.

The splenic artery most often originates from the trifurcation of the celiac trunk and less often forms the gastrosplenic trunk.

The inner vascular structure of the spleen was also studied. The splenic artery within an organ is usually divided into two or three branches, which further form a complex network of arterial vessels. A similar confluence (similar to branching) is observed in the venous vasculature. Understanding the specific characteristics of the intra‐organ splenic vasculature is important in spleen surgeries because this approach allows us to minimize the risk of bleeding and preserve the maximum possible amount of healthy tissue in the organ.

Finally, we would like to emphasize the need for further research in this area for a comprehensive understanding of the splenic anatomy and vasculature, which is especially important for performing surgical interventions in the upper abdominal cavity.

## Author Contributions

**Ilias Miltiadis:** conceptualization, investigation, writing – original draft, writing – review and editing, supervision, visualization, methodology. **Edgar S. Kafarov:** conceptualization, investigation, methodology. **Ali S. Dadashev:** conceptualization, investigation, methodology. **Pavel Burko:** conceptualization, investigation, writing – original draft, writing – review and editing, visualization. **Daria A. Sukmanova:** investigation, validation. **Nadezhda A. Stashevskaya:** investigation, validation. **Oleg K. Zenin:** project administration, resources, formal analysis.

## Funding

The authors have nothing to report.

## Ethics Statement

The authors have nothing to report.

## Consent

The authors have nothing to report.

## Conflicts of Interest

The authors declare no conflicts of interest.

## Transparency Statement

The lead/Corresponding author (Ilias Miltiadis) affirms that this manuscript is an honest, accurate, and transparent account of the study being reported; that no important aspects of the study have been omitted; and that any discrepancies from the study as planned (and, if relevant, registered) have been explained.

## Supporting information


**Figure S1:** Forest plots displaying the random‐effects pooled mean estimates for (a) splenic length, (b) splenic width, and (c) splenic thickness.

**Figure S2:** (a) Random‐effects meta‐analysis of overall pooled splenic volume (cm3), including subgroup stratifications by imaging and anatomical modality. (b) Continuous two‐group meta‐analysis demonstrating the mean difference (MD) in splenic volume between male and female cohorts.

**Figure S3:** Forest plot of the pooled mean splenic artery diameter (mm).

**Figure S4:** (a) Proportional meta‐analysis illustrating the pooled global prevalence of exactly two primary terminal splenic artery branches. (b) Proportional meta‐analysis illustrating the pooled prevalence of exactly three primary terminal branches. Proportions were calculated using the Freeman‐Tukey double arcsine transformation.

Supporting File 1

## Data Availability

The data that support the findings of this study are openly available in Github at https://github.com/iliasmiltiadis/MA-spleen.
