# Supplementary figures and images for: The Roadmap of the Spleen: A Meta‐Analysis of Morphometric and Vascular Anatomy
Source: Health Sci Rep. 2026 Jun 18;9(6):e72667. doi: 10.1002/hsr2.72667 (PMC13277743; doi:10.1002/hsr2.72667)

## a Splenic length (cm)

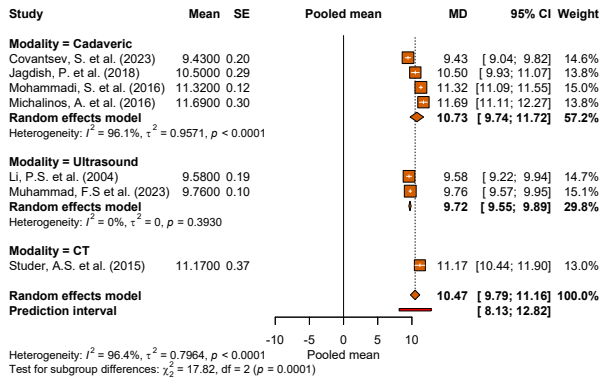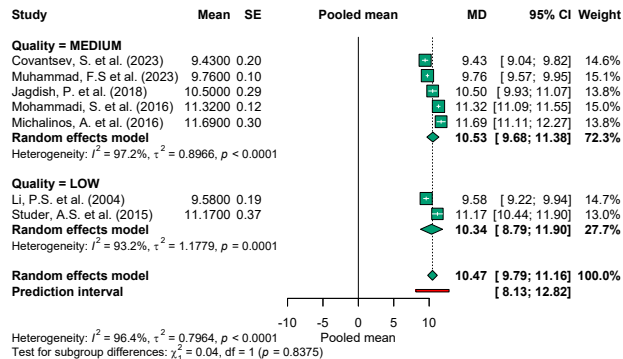

## b Splenic width (cm)

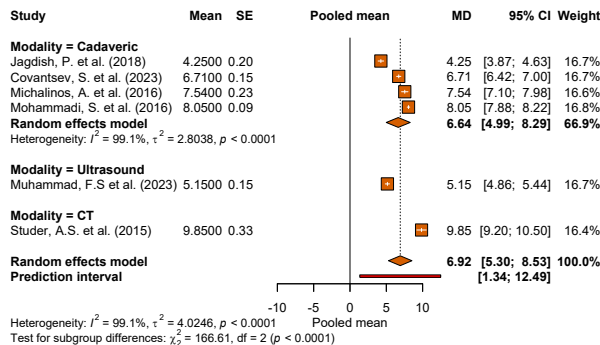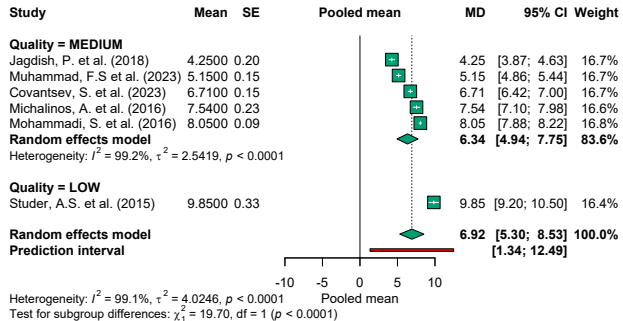

## c Splenic thickness (cm)

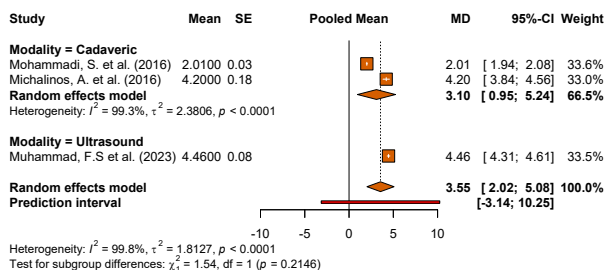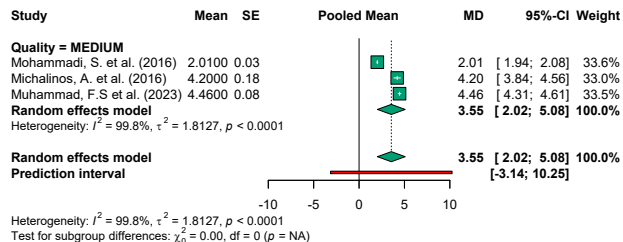

Supplement: Supplementary file 1 — Figure S1: Forest plots displaying the random‐effects pooled mean estimates for (a) splenic length, (b) splenic width, and (c) splenic thickness. [file HSR2-9-e72667-s003.pdf]

## a Splenic volume (cm<sup>3</sup>)

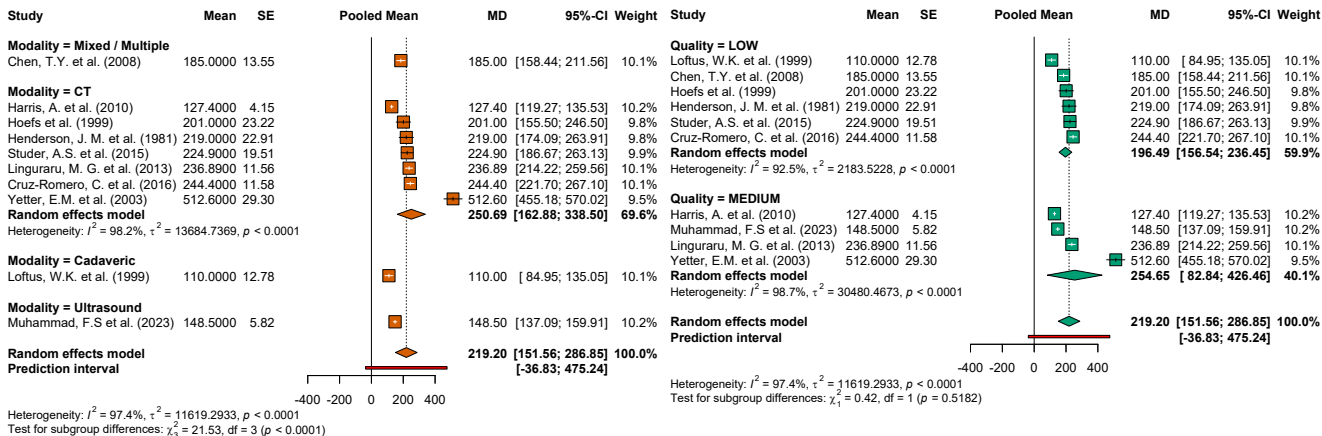

## b Mean difference in splenic volume between male and female cohorts

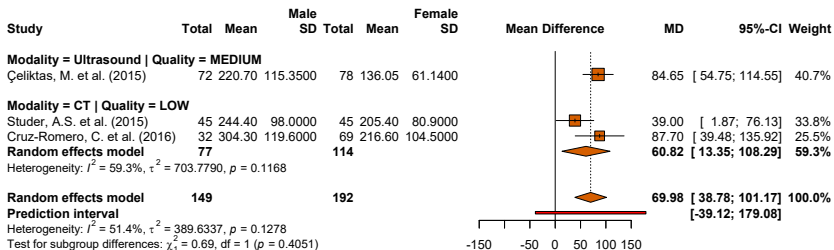

Supplement: Supplementary file 2 — Figure S2: (a) Random‐effects meta‐analysis of overall pooled splenic volume (cm3), including subgroup stratifications by imaging and anatomical modality. (b) Continuous two‐group meta‐analysis demonstrating the mean difference (MD) in splenic volume between male and female cohorts. [file HSR2-9-e72667-s005.pdf]

# Splenic artery diameter (mm)

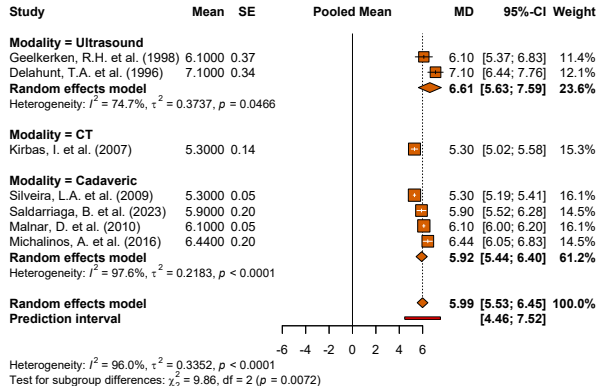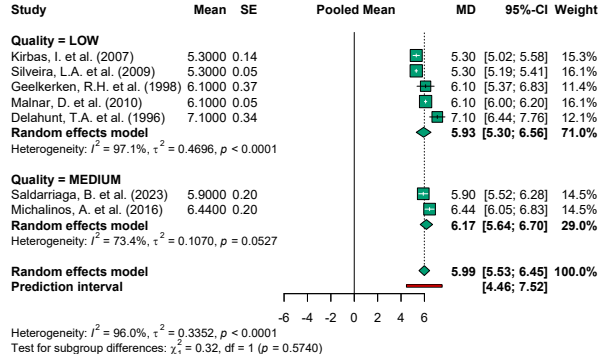

Supplement: Supplementary file 3 — Figure S3: Forest plot of the pooled mean splenic artery diameter (mm). [file HSR2-9-e72667-s001.pdf]
