# Supplementary material for: The Roadmap of the Spleen: A Meta‐Analysis of Morphometric and Vascular Anatomy
Source: Health Sci Rep. 2026 Jun 18;9(6):e72667. doi: 10.1002/hsr2.72667 (PMC13277743; doi:10.1002/hsr2.72667)
Supplement: Supplementary file 4 — Figure S4: (a) Proportional meta‐analysis illustrating the pooled global prevalence of exactly two primary terminal splenic artery branches. (b) Proportional meta‐analysis illustrating the pooled prevalence of exactly three primary terminal branches. Proportions were calculated using the Freeman‐Tukey double arcsine transformation. [file HSR2-9-e72667-s002.pdf]

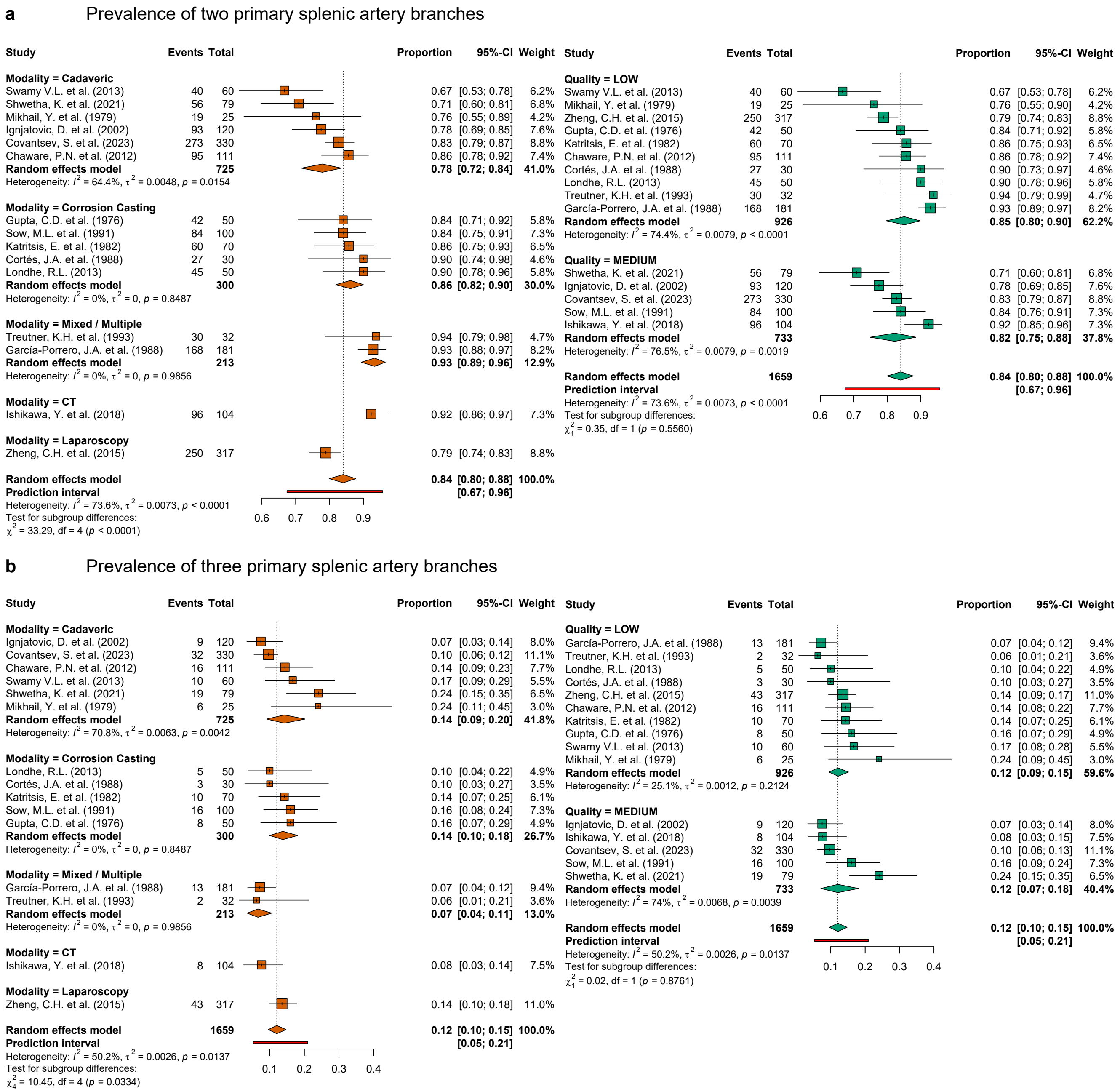
a
Prevalence of two primary splenic artery branches
Study Events Total Proportion 95%-CI Weight
Modality = Cadaveric
Swamy V.L. et al. (2013) 40 60 0.67 [0.53; 0.78] 6.2%
Shwetha, K. et al. (2021) 56 79 0.71 [0.60; 0.81] 6.8%
Mikhail, Y. et al. (1979) 19 25 0.76 [0.55; 0.89] 4.2%
Ignjatovic, D. et al. (2002) 93 120 0.78 [0.69; 0.85] 7.6%
Covantsev, S. et al. (2023) 273 330 0.83 [0.79; 0.87] 8.8%
Chaware, P.N. et al. (2012) 95 111 0.86 [0.78; 0.92] 7.4%
Random effects model 725 0.78 [0.72; 0.84] 41.0%
Heterogeneity: I^2 = 64.4%, τ^2 = 0.0048, p = 0.0154
Modality = Corrosion Casting
Gupta, C.D. et al. (1976) 42 50 0.84 [0.71; 0.92] 5.8%
Sow, M.L. et al. (1991) 84 100 0.84 [0.75; 0.91] 7.3%
Katritsis, E. et al. (1982) 60 70 0.86 [0.75; 0.93] 6.5%
Cortés, J.A. et al. (1988) 27 30 0.90 [0.74; 0.98] 4.6%
Londhe, R.L. (2013) 45 50 0.90 [0.78; 0.96] 5.8%
Random effects model 300 0.86 [0.82; 0.90] 30.0%
Heterogeneity: I^2 = 0%, τ^2 = 0, p = 0.8487
Modality = Mixed / Multiple
Treutner, K.H. et al. (1993) 30 32 0.94 [0.79; 0.98] 4.7%
García-Porrero, J.A. et al. (1988) 168 181 0.93 [0.88; 0.97] 8.2%
Random effects model 213 0.93 [0.89; 0.96] 12.9%
Heterogeneity: I^2 = 0%, τ^2 = 0, p = 0.9856
Modality = CT
Ishikawa, Y. et al. (2018) 96 104 0.92 [0.86; 0.97] 7.3%
Modality = Laparoscopy
Zheng, C.H. et al. (2015) 250 317 0.79 [0.74; 0.83] 8.8%
Random effects model 0.84 [0.80; 0.88] 100.0%
Prediction interval [0.67; 0.96]
Heterogeneity: I^2 = 73.6%, τ^2 = 0.0073, p < 0.0001
Test for subgroup differences:
χ^2 = 33.29, df = 4 (p < 0.0001)
0.6 0.7 0.8 0.9
Study Events Total Proportion 95%-CI Weight
Quality = LOW
Swamy V.L. et al. (2013) 40 60 0.67 [0.53; 0.78] 6.2%
Mikhail, Y. et al. (1979) 19 25 0.76 [0.55; 0.90] 4.2%
Zheng, C.H. et al. (2015) 250 317 0.79 [0.74; 0.83] 8.8%
Gupta, C.D. et al. (1976) 42 50 0.84 [0.71; 0.92] 5.8%
Katritsis, E. et al. (1982) 60 70 0.86 [0.75; 0.93] 6.5%
Chaware, P.N. et al. (2012) 95 111 0.86 [0.78; 0.92] 7.4%
Cortés, J.A. et al. (1988) 27 30 0.90 [0.73; 0.97] 4.6%
Londhe, R.L. (2013) 45 50 0.90 [0.78; 0.96] 5.8%
Treutner, K.H. et al. (1993) 30 32 0.94 [0.79; 0.99] 4.7%
García-Porrero, J.A. et al. (1988) 168 181 0.93 [0.89; 0.97] 8.2%
Random effects model 926 0.85 [0.80; 0.90] 62.2%
Heterogeneity: I^2 = 74.4%, τ^2 = 0.0079, p < 0.0001
Quality = MEDIUM
Shwetha, K. et al. (2021) 56 79 0.71 [0.60; 0.81] 6.8%
Ignjatovic, D. et al. (2002) 93 120 0.78 [0.69; 0.85] 7.6%
Covantsev, S. et al. (2023) 273 330 0.83 [0.79; 0.87] 8.8%
Sow, M.L. et al. (1991) 84 100 0.84 [0.76; 0.91] 7.3%
Ishikawa, Y. et al. (2018) 96 104 0.92 [0.85; 0.96] 7.3%
Random effects model 733 0.82 [0.75; 0.88] 37.8%
Heterogeneity: I^2 = 76.5%, τ^2 = 0.0079, p = 0.0019
Random effects model 1659 0.84 [0.80; 0.88] 100.0%
Prediction interval [0.67; 0.96]
Heterogeneity: I^2 = 73.6%, τ^2 = 0.0073, p < 0.0001
Test for subgroup differences:
χ^2_1 = 0.35, df = 1 (p = 0.5560)
0.6 0.7 0.8 0.9
b
Prevalence of three primary splenic artery branches
Study Events Total Proportion 95%-CI Weight
Modality = Cadaveric
Ignjatovic, D. et al. (2002) 9 120 0.07 [0.03; 0.14] 8.0%
Covantsev, S. et al. (2023) 32 330 0.10 [0.06; 0.12] 11.1%
Chaware, P.N. et al. (2012) 16 111 0.14 [0.09; 0.23] 7.7%
Swamy V.L. et al. (2013) 10 60 0.17 [0.09; 0.29] 5.5%
Shwetha, K. et al. (2021) 19 79 0.24 [0.15; 0.35] 6.5%
Mikhail, Y. et al. (1979) 6 25 0.24 [0.11; 0.45] 3.0%
Random effects model 725 0.14 [0.09; 0.20] 41.8%
Heterogeneity: I^2 = 70.8%, τ^2 = 0.0063, p = 0.0042
Modality = Corrosion Casting
Londhe, R.L. (2013) 5 50 0.10 [0.04; 0.22] 4.9%
Cortés, J.A. et al. (1988) 3 30 0.10 [0.03; 0.27] 3.5%
Katritsis, E. et al. (1982) 10 70 0.14 [0.07; 0.25] 6.1%
Sow, M.L. et al. (1991) 16 100 0.16 [0.08; 0.24] 7.3%
Gupta, C.D. et al. (1976) 8 50 0.16 [0.07; 0.29] 4.9%
Random effects model 300 0.14 [0.10; 0.18] 26.7%
Heterogeneity: I^2 = 0%, τ^2 = 0, p = 0.8487
Modality = Mixed / Multiple
García-Porrero, J.A. et al. (1988) 13 181 0.07 [0.04; 0.12] 9.4%
Treutner, K.H. et al. (1993) 2 32 0.06 [0.01; 0.21] 3.6%
Random effects model 213 0.07 [0.04; 0.11] 13.0%
Heterogeneity: I^2 = 0%, τ^2 = 0, p = 0.9856
Modality = CT
Ishikawa, Y. et al. (2018) 8 104 0.08 [0.03; 0.14] 7.5%
Modality = Laparoscopy
Zheng, C.H. et al. (2015) 43 317 0.14 [0.10; 0.18] 11.0%
Random effects model 1659 0.12 [0.10; 0.15] 100.0%
Prediction interval [0.05; 0.21]
Heterogeneity: I^2 = 50.2%, τ^2 = 0.0026, p = 0.0137
Test for subgroup differences:
χ^2_4 = 10.45, df = 4 (p = 0.0334)
0.1 0.2 0.3 0.4
Study Events Total Proportion 95%-CI Weight
Quality = LOW
García-Porrero, J.A. et al. (1988) 13 181 0.07 [0.04; 0.12] 9.4%
Treutner, K.H. et al. (1993) 2 32 0.06 [0.01; 0.21] 3.6%
Londhe, R.L. (2013) 5 50 0.10 [0.04; 0.22] 4.9%
Cortés, J.A. et al. (1988) 3 30 0.10 [0.03; 0.27] 3.5%
Zheng, C.H. et al. (2015) 43 317 0.14 [0.09; 0.17] 11.0%
Chaware, P.N. et al. (2012) 16 111 0.14 [0.08; 0.22] 7.7%
Katritsis, E. et al. (1982) 10 70 0.14 [0.07; 0.25] 6.1%
Gupta, C.D. et al. (1976) 8 50 0.16 [0.07; 0.29] 4.9%
Swamy V.L. et al. (2013) 10 60 0.17 [0.08; 0.28] 5.5%
Mikhail, Y. et al. (1979) 6 25 0.24 [0.09; 0.45] 3.0%
Random effects model 926 0.12 [0.09; 0.15] 59.6%
Heterogeneity: I^2 = 25.1%, τ^2 = 0.0012, p = 0.2124
Quality = MEDIUM
Ignjatovic, D. et al. (2002) 9 120 0.07 [0.03; 0.14] 8.0%
Ishikawa, Y. et al. (2018) 8 104 0.08 [0.03; 0.15] 7.5%
Covantsev, S. et al. (2023) 32 330 0.10 [0.06; 0.13] 11.1%
Sow, M.L. et al. (1991) 16 100 0.16 [0.09; 0.24] 7.3%
Shwetha, K. et al. (2021) 19 79 0.24 [0.15; 0.35] 6.5%
Random effects model 733 0.12 [0.07; 0.18] 40.4%
Heterogeneity: I^2 = 74%, τ^2 = 0.0068, p = 0.0039
Random effects model 1659 0.12 [0.10; 0.15] 100.0%
Prediction interval [0.05; 0.21]
Heterogeneity: I^2 = 50.2%, τ^2 = 0.0026, p = 0.0137
Test for subgroup differences:
χ^2_1 = 0.02, df = 1 (p = 0.8761)
0.1 0.2 0.3 0.4
