# Supplementary material for: The Roadmap of the Spleen: A Meta‐Analysis of Morphometric and Vascular Anatomy
Source: Health Sci Rep. 2026 Jun 18;9(6):e72667. doi: 10.1002/hsr2.72667 (PMC13277743; doi:10.1002/hsr2.72667)
Supplement: Supplementary file 5 — Supporting File 1 [file HSR2-9-e72667-s004.docx]

**Supplementary Table S1.** Main characteristics of the studies included in the review

| **Study** | **Year** | **Location** | **Population** | **Study method** | **Sample size (N)** |
| --- | --- | --- | --- | --- | --- |
| Muhammad, F.S et al. (2023) | 2023 | Lahore, Pakistan | 100 pregnant and 100 nonpregnant women, mean age 29 ± 7.8 years. | Ultrasound | 200 |
| Türkyılmaz, Z. et al (2023) | 2023 | Edrine, Türkiye | ♂ – 567  ♀ – 374  Average age – 61.07±13.79 | Contrast-enhanced abdominal CT | 941 |
| Covantsev, S. et al. (2023) | 2023 | Chisinau, Moldova |  | Cadaver study | 330 |
| Saldarriaga, B. et al. (2023) | 2023 | Bucaramanga, Colombia | Mixed race men aged 20-65 | Cadaver study | 26 |
| Russkikh, A.N. (2022) | 2022 | Krasnoyarsk, Russia | 53 men, average age 54.9 years, range 36 to 71 years | 3D models of the portal system based on multislice contrast-enhanced CT | 53 |
| Acar, G. et al. (2022) | 2022 | Türkiye | Patients without surgical intervention, pathologies and diseases aged from 18 to 82, average age 61.7±15.3 y.o.  ♂ – 214  ♀ – 126 | Contrast CT scan of the abdomen | 340 |
| Laleye, C.M. et al. (2021) | 2021 | Cotonou, Benin | People aged 13 to 87 years, average age 51.44±16.35 years | MRI | 200 |
| Shwetha, K. et al. (2021) | 2021 | Karnataka, India | Population of Mysore | Cadaver study | 79 |
| Khaleel, N. et al. (2021) | 2021 | Ethiopia, Africa,  Karnataka, India |  | Cadaver study | 100 |
| Brinkman, D.J. et al. (2020) | 2020 | Eindhoven, Netherlands | People aged 30 to 69 years.  ♂ – 40  ♀ – 40 | Retrospective, cohort study using contrast -enhanced CT | 80 |
| Fataftah, J. et al. (2020) | 2020 | Jordan, Minnesota, USA | People range in age from 18 to 82, with an average age of 53.  ♂ – 114  ♀ – 105 | Abdominal contrast -enhanced CT | 219 |
| Wada, Y. et al. (2020) | 2020 | Tokyo, Japan | Men aged from 19 to 86 years. | 3D CT | 30 |
| Ekingen, A. et al. (2020) | 2020 | Türkiye | People ranged in age from 16 to 93 years, mean age 50.6 ± 16.2 years.  ♂ – 406  ♀ – 344 | CT | 750 |
| Sundar, G. et al. (2020) | 2020 | Vellore, India |  | Cadaver study | 60 |
| Ishikawa, Y. et al. (2018) | 2018 | Japan | People range in age from 43 to 89 years, with a median age of 72 years. | 3D CT | 104 |
| Zhu, C. et al. (2018) | 2018 | Seoul, South Korea |  | 3D CT | 169 |
| Pinal-Garcia, D.F. et al. (2018) | 2018 | Mexico | People aged from 18 to 79 years old, with an average age of 48.8 years.  ♂ – 121  ♀ – 19 | Cadaver study | 140 |
| Jagdish, P. et al. (2018) | 2018 | India |  | Cadaver study | 60 |
| Meet, K. et al. (2017) | 2017 | Jakarta, Indonesia | ♂ – 11  ♀ – 4 | Cadaver study | 15 |
| Ashok, K.R. et al. (2016) | 2016 | India |  | Cadaver study | 76 |
| Cruz-Romero, C. et al. (2016) | 2016 | Boston, USA | ♂ – 32  ♀ – 69 | Retrospective study, abdominal CT | 101 |
| Michalinos, A. et al. (2016) | 2016 | Athens, Greece | Caucasians, average age 65.9±21.4 years.  ♂ – 34  ♀ – 16 | Cadaver study | 50 |
| Mohammadi, S. et al. (2016) | 2016 | Mashhad, Iran | ♂ – 541  ♀ – 152  Average age 40.28 ± 20.97 years. | Cadaver study | 693 |
| Osman, A.M. et al. (2016) | 2016 | Cairo, Egypt |  | CT | 1000 |
| Inoko, K. et al. (2015) | 2015 | Hokkaido, Japan | People aged 37 to 80, average age 70 | Laparoscopy | 6 |
| Zheng, C.H. et al. (2015) | 2015 | Fujian, China | Patients who have undergone total gastrectomy | Laparoscopy | 317 |
| Studer, A.S. et al. (2015) | 2015 | Marseille, France | Nonobese patients over 16 years of age | Retrospective study based on CT data | 90 |
| Çeliktas, M. et al. (2015) | 2015 | Adana, Turkey | 78 men and 72 women aged 18 to 76 years | Ultrasound | 150 |
| Torres, K. et al. (2015) | 2015 | Lublin, Poland | ♂ – 740  ♀ – 829  People aged 16 to 96, average age 58 | CT | 1569 |
| Araujo Neto, S.A. et al. (2015) | 2015 | Joao Pessoa, Brazil | ♂ –25  ♀ – 35 | CT | 60 |
| Gangadhara, R.P. et al. (2014) | 2014 | Chittoor, India |  | Cadaver study | 30 |
| Linguraru, M. G. et al. (2013) | 2013 | Washington, USA | ♂ – 115, ages 17 to 76, average age 45.5  ♀ – 57 aged 18 to 74 years, average age 47.5 | CT | 172 |
| Londhe, R.L. (2013) | 2013 | Karnataka, India |  | Corrosion casting | 50 |
| Swamy V.L. et al. (2013) | 2013 | Anhara Pradesh, India _ |  | Cadaver study | 60 |
| Chaware, P.N. et al. (2012) | 2012 | Nagpur, India | ♂ – 96  ♀ – 15 | Cadaver study | 111 |
| Krumm, P. et al. (2011) | 2011 | Tübingen, Germany | Age – 18-94,  Average age – 59.3 | Abdominal contrast -enhanced CT | 916 |
| Singh, A. et al. (2011) | 2011 | Bihari population, India | People aged 20 to 60 years.  ♂ – 80  ♀ – 80 | Ultrasound | 160 |
| Harris, A. et al.(2010) | 2010 | Sapporo, Japan | People aged 20 to 85 years, mean age 48.7 ± 16.1  ♂ – 113  ♀ – 117 | CT | 130 |
| Malnar, D. et al. (2010) | 2010 | Rijeka, Croatia | People aged 35 to 70 years | Cadaver study | 90 |
| Xu, W.-L. et al. (2009b) | 2009 | Hebei, China | People aged 1.5 to 25 years, average age 9.21 ± 4.06 years)  ♂ – 21  ♀ – 15 | Color Dopplerography | 36 |
| Xu, W.L. et al. (2009a) | 2009 | Shijiazhuang, China |  | Color Dopplerography | 48 |
| Silveira, L.A. et al. (2009) | 2009 | Sao Paulo, Brazil | ♂ – 21 | Cadaver study | 21 |
| Chen, T.Y. et al. (2008) | 2008 | Taiwan, China | People aged 22 to 54 years, average age 33.8 ± 8.4 years  ♂ – 15  ♀ – 8 | Ultrasound  CT | 23 |
| Kirbas, I. et al. (2007) | 2007 | Ankara, Türkiye | Adults aged 7 to 39 years, average age 24  ♂ – 197  ♀ – 1 | CT | 198 |
| Li, P.S. et al. (2004) | 2004 | Hong Kong, China | Adults aged from 30 to 59 years (average age – 44.5 years) | Ultrasound | 43 |
| Pandey, S.K. et al. (2004) | 2004 | Varanasi, India | ♂ – 264  ♀ – 56 | Cadaver study | 320 |
| Sahni, A.D. et al. (2003) | 2003 | Chandigarh, India | People aged 18 to 80 years.  ♂ – 156  ♀ – 44 | Cadaver study | 200 |
| Yetter, E.M. et al. (2003) | 2003 | Kansas City, USA | ♂ – 66  ♀ – 51 | CT | 142 |
| Ignjatovic, D. et al. (2002) | 2002 | Ford, Norway | People aged 26 to 83, average age 54  ♂ – 53  ♀ – 49 | Cadaver study | 102 |
| Loftus, W.K. et al. (1999) | 1999 | South Australia | People aged 30 to 86 years, average age 65 ± 16.5 years  ♂ – 17  ♀ – 13 | Cadaver study | 30 |
| Hoefs et al. (1999) | 1999 | California, USA |  | SPECT scan | 443 |
| Geelkerken, R.H. et al. (1998) | 1998 | Enschede, Netherlands | People aged 39 to 81, average age 65  ♂ – 12  ♀ – 2 | Angiography  Duplex ultrasonography | 14 |
| Prassopoulos, P. et al. (1997) | 1997 | Heraklion, Greece | People aged 20 and older.  ♂ – 73  ♀ – 67 | CT | 140 |
| Delahunt, T.A. et al. (1996) | 1996 | Enschede, Netherlands | People aged 39 to 81, average age 65 | Duplex sonography | 25 |
| Sylvester, P.A. et al. (1995) | 1995 | Cambridge, UK |  | Cadaver study, angiography | 73 |
| Gómez Pellico, L. et al. (1994) | 1994 | Spain | Men – 23  Women – 9. Participants aged from 3 to 86. Average age – 42 ± 24 | Corrosion casts | 32 |
| Treutner, K.H. et al. (1993) | 1993 | Aachen, Germany | ♂ – 16  ♀ – 16 | Angiography and corrosion casting | 32 |
| Sow, M.L. et al. (1991) | 1991 | Dakar, Senegal | Africans of various ages and both sexes | Corrosion casting | 100 |
| Cortés, J.A. et al. (1988) | 1988 | Madrid, Spain | People range in age from 10 to 88 years, with an average age of 63.5 years.  ♂ – 18  ♀ – 12 | Corrosive casting | 30 |
| García-Porrero, J.A. et al. (1988) | 1988 | Santander, Spain | ♂ – 33  ♀ – 48 | Angiography and corrosion casting | 181 |
| Katritsis, E. et al. (1982) | 1982 | Athens, Greece |  | Angiography,  corrosive casting | 70 |
| Henderson, J. M. et al. (1981) | 1981 | Atlanta, USA | Patients aged 20-30 years, without liver disease | CT | 11 |
| Mikhail, Y. et al. (1979) | 1979 | Cairo, Egypt |  | Cadaver study | 25 |
| Gupta, C.D. et al. (1976) | 1976 | Agra, India | Adults | Corrosive casting | 50 |
